# The Effectiveness of Wogonin on Treating Cough Mice With Mycoplasma Pneumoniae Infection

**DOI:** 10.3389/fmolb.2022.803842

**Published:** 2022-07-15

**Authors:** Mingchuan Liang, Yanli Meng, Xiaoxi Wang, Lei Wang, Guixin Tang, Weiming Wang

**Affiliations:** ^1^ Heilongjiang Academy of Chinese Medicine Sciences, Harbin, China; ^2^ Advanced Microscopy and Instrumentation Research Center, Harbin Institute of Technology, Harbin, China; ^3^ School of Chinese Medicine, Southern Medical University, Guangzhou, China

**Keywords:** wogonin, biacore, cough, mycoplasma pneumoniae, TRPA1

## Abstract

**Background:** Cough is the main symptom of mycoplasma pneumoniae (MP) infection. Cough potential protein transient receptor potential A1 (TRPA1) plays an important role in cough reflex. The purpose of this study was to clarify the mechanism of wogonin, the effective component of Qinbai Qingfei concentrated pellet (Qinbai), in the treatment of cough after MP infection.

**Methods:** The Biacore™ system was used to detect whether there was specific binding between Qinbai and cough potential protein TRPA1. Biacore™ fishing technology and UPLC-Q-TOF-MS technology were used during fishing combined active components and identification and analysis of recovered samples. The expression levels of TRPA1, substance P (SP), calcitonin gene-related peptide (CGRP), cough-related proteins, and mRNA in the lung tissues from each group were detected by immunohistochemistry, Western blot, and real-time PCR.

**Results:** Biacore™ results showed that Qinbai had strong specific binding to TRPA1 protein with a binding value of 99.0 resonance unit (RU). The samples obtained from angling were identified and analyzed by UPLC-Q-TOF-MS as wogonin. The results of immunohistochemistry, Western blot, and real-time PCR showed that compared with the model group, the wogonin group had lower expressions of mRNA, TRPA1, SP, and CGRP in the lung tissue of cough mice with MP infection (*p* < 0.01 or *p* < 0.05), and the effects were superior to those of azithromycin and pentoxyverine control groups.

**Conclusion:** Wogonin can treat cough after MP infection by affecting the expressions of cough-related proteins, such as TRPA1, SP, and CGRP. This study provided a theoretical foundation for the clinical research of Qinbai.

## 1 Introduction

Mycoplasma pneumoniae (MP) is the smallest anaerobic microorganism between bacteria and virus that can live independently, and it is transmitted through the respiratory tract. The clinical manifestations of MP are mainly repeated fever, stimulating dry cough, headache, and sore throat (Kim et al., 2019). Cough after MP infection is the main respiratory symptom, and persistent dry cough or cough with small amount of sputum is one of the main characteristics (Megumi et al., 2020). Autumn and winter are the peak seasons of this disease. In recent years, the clinical incidence rate has been increasing, which may lead to bronchiectasis and pulmonary fibrosis, or even death in severe situation, if not treated in time (Meng et al., 2019). β-Lactamase antibiotics such as penicillin and cephalosporin are ineffective in treating MP infection due to the lack of cell wall structure in MP (Xu et al., 2018). Macrolide antibiotic azithromycin is effective in treating MP infection in children, but clinical studies have shown that long-term intravenous infusion of azithromycin will increase the incidence of adverse reactions, such as resistance, gastrointestinal reactions, local pain, and anorexia (Ma et al., 2017). Therefore, it is particularly important to find a drug that can treat MP, alleviate the symptoms of cough after infection, and reduce side effects.

In recent years, the study of the treatment of mycoplasma pneumonia in Chinese medicine has made great progress. Qinbai Qingfei concentrated pellet (Qinbai) is the first new Chinese medicine developed by Heilongjiang Academy of Chinese Medicine to treat mycoplasma pneumonia in children. It is prepared from scutellaria root, stemona root, aster root, platycodon root, earthworm, and ophiopogon root. Meng Yanli et al. demonstrated that Qinbai could reduce the expression of pulmonary fibrosis factors β-catenin and α-SMA in model mice and inhibit the interstitial pneumonia caused by MP (Liang et al., 2017). Yao et al. (2016) found that Qinbai could inhibit the EMT of AEC-Ⅱ and increase the expression of SP-A by reducing the expression of TGF-β, which played a role in the protection and repair of lung tissue. Wogonin is the main component of scutellaria. Wogonin has the pharmacological effects of antivirus, antitumor and anti-inflammation (Zhuang et al., 2020), and has a good inhibitory effect on a variety of pro-inflammatory factors.

Surface plasmon resonance (SPR) measures the change in optical parameters caused by the interaction between receptors and ligands to determine whether protein–protein molecules interact at the molecular level (Li et al., 2016). SPR technology has the advantage of simple, rapid, and real-time detection of the dynamic process of the reaction. It has been widely used in the research of drugs and proteins. SPR biosensors, such as the Biacore™ system, have become standard tools for protein characterization and binding interaction studies. Herbal medicine has complex components, often containing dozens or even hundreds of components, which makes it very difficult to find the binding relationship between the active components and the protein. The measurement of such binding relationship is made possible by Biacore™.

Transient receptor potential A1 (TRPA1), an important member of TRP family, is mainly distributed in C fibers. C fibers are distributed almost throughout the respiratory system. In recent years, studies have shown that TRPA1 is the key protein (Dong et al., 2020) of cough reflex. TRPA1 plays an important role in respiratory diseases, and most compounds that induce cough can activate the TRPA1 channel. The activation of TRPA1 can cause Ca2^+^ influx, which causes nerve endings to release tachykinin, calcitonin gene-related peptide (CGRP) and substance P (SP), and induce inflammatory reactions. Therefore, whether the TRPA1 channel is inhibited becomes important for the cough treatment after MP infection. In this study, the expression levels of TRPA1, SP, and CGRP were detected by Biacore™, immunohistochemistry, Western blot, and real-time PCR. This article studied the mechanism of wogonin, an active ingredient in Qinbai, in the treatment of cough after MP infection.

## 2 Materials

### 2.1 Ethical Statement

All animal experiments in this study were carried out in strict accordance with the “Guideline for the Care and Use of Experimental Animals in Heilongjiang Province.” “Protocol of Animal Protection and Utilization Committee of Heilongjiang Academy of Chinese Medicine” was approved by Animal Management Committee of Heilongjiang Province [License No. (2011)93]. All operations were performed under sodium chloral hydrate anesthesia to reduce the pain of laboratory animals, and the mice were humanely killed by cervical dislocation.

### 2.2 Reagents

The following reagents were used in this experiment: peroxidase-bound goat antirabbit IgG (H + L) (China Beijing Zhongshan Jinqiao Biological Company, Lot No. 210060330), high-sensitivity ECL chemiluminescence kit (China Wanlei biology science and technology company, Lot No. 30A041), rabbit anti-β-actin (bs-0061R) antibody (China Bioss company, Lot No. AH11286487), TRPA1 protein (Proteintech Company of America, Lot No. P20200629061), TRIzol (Invitrogen Company of America, Lot No. 19124-1-AP), SDS-PAGE gel preparation kit (Beijing Soleibao Biological Company, Lot No. 2021: 0421), PVDF membrane (American Millipore company, Lot No. 21183776), BCA protein detection kit (Beijing Soleibao Biological Company, Lot No. 20201010, China), UltraSYBR One-Step RT-qPCR kit (Beijing CWBIO biology science and technology company, Lot No. 29520), CM5 chips (American GE Healthcare company, Lot No. 10280148).

All primers were synthesized by Shanghai Bioengineering Technology Co., Ltd.

### 2.3 Experimental Animals

Twenty 6-week male and twenty 6-week female BALB/c mice of SPF grade weighing 20–22 g were purchased from Harbin Medical University and reared at room temperature 25°C and humidity 60% in the GLP laboratory [Animal Certificate No. SCXK (Black) 2019-001].

### 2.4 Experimental Drugs

Qinbai, provided by China Harbin Tianheli Pharmaceutical Co., Ltd. (Lot No. CXZS1000045). The wogonin reference standard was provided by Heilongjiang Institute for Drug Control (Lot No. H01811804026).

## 3 Methods

### 3.1 Preparation of Wogonin Solution

Wogonin reference substance (1 mg) added into nuclease-free water to prepare a 10 mg/ml solution. The resulted solution was filtered and stored at −20°C for subsequent use.

### 3.2 Biacore™ Test

#### 3.2.1 Pre-Enrichment Test

Based on the Biacore™ T200 test system, TRPA1 protein was coupled to channel 2, and channel 1 was used as a reference channel. TRPA1 protein (200 mg/ml) were weighed and added to 97 uL acetate buffers with pH 40, 4.5, and 5.0, respectively to prepare protein solutions. The resulted mixture was placed in an uncovered 1.5 ml Eppendorf (EP) tube. Another 300 μl NaOH solution was placed in a separate EP tube for chip regeneration. The protein solution was injected into the system at 10 μl/min at 25°C, and the injection was completed in 60 s. After incubated for another 10 s, the resulted chip was placed in 0.05 mol/L NaOH for 30 s for regeneration. The prepared three protein solutions were tried separately to determine the optimal coupling condition. The resonance unit (RU) value of the coupling of TRPA1 protein to the chip was measured.

#### 3.2.2 Ligand Coupling Test

Then, 140 μl ethanolamine solution, 100 μl EDC solution, and 100 μl NHS solution were placed at the designated positions on the reagent rack. EDC and NHS were used to activate the carboxyl group on the chip surface. According to the results from pre-enrichment test, sodium acetate solution with pH 4.0 was selected as the optimal coupling condition, and 200 μl protein solution was prepared accordingly with 194 μl sodium acetate buffer and 6 μl TRPA1 protein (200 mg/ml). The resulted protein solution was added to the uncovered EP tube. The protein solution was injected onto the chip surface by pulse injection at a flow rate of 10 μl/min for the coupling time of 600 s. After the remaining carboxyl groups on the CM5 chip surface were blocked with ethanolamine solution, the loosely bound TRPA1 protein was further rinsed off with PBS.

#### 3.2.3 Test of Binding Ability Between Qinbai and Transient Receptor Potential A1 Protein

Qinbai was dissolved in PBS buffer to make 20 mg/ml Qinbai solution, and the resulted solution was then filtered. The sample was injected at 30 μl/min over the chip surface for 60 s. The regeneration reagent was glycine–hydrochloric acid buffer pH 2.0, and the regeneration time was 30 s. The sample detection temperature was set at 25°C. The binding force of Qinbai to the target protein TRPA1 was determined by measuring the RU value in the sensor map.

#### 3.2.4 Fishing Affinity Components by the Biacore™ Fishing Method

The prepared Qinbai solution (20 mg/ml) was injected onto the surface of CM5 chip at a flow rate of 5 μl/min and the coupling time of 180 s. The unbound sample solution was rinsed off with PBS buffer. After 2 μl of sample recovery solution (0.5% HCOOH) was injected into the flow cell for 20 s incubation, the combination of Qinbai and TRPA1 was decomposed and separated into sample recovery solution. Then, the flow direction of the sample on the surface of the sensor was changed to the opposite direction to deposit the recovery solution, containing the binding components in 10 μl of NH_4_HCO_3_ (50 mmol/L). The cycle number for this process was set at 20 so that sufficient angling product was recovered for mass spectrometric detection.

### 3.3 Analysis of Fishing Products by UPLC-Q-TOF-MS

#### 3.3.1 Preparation of Solution

Samples of Biacore™ fishing were dried using a nitrogen blower. The dried samples were dissolved in 100 μl methanol, centrifuged at 12,000 rpm for 20 min at 4°C, and the supernatant was taken for subsequent experiments.

#### 3.3.2 Chromatographic Condition

The chromatographic analysis used the Waters ACQUITY^TM^ BEH C_18_ column (2.1 mm × 100 mm, 1.7 μm) and BEH C_18_ VanGuard Pre-column (100 mm × 2.1 mm, 1.7 μm). Column temperature was maintained constantly at 30°C, with 0.1% formic acid aqueous solution (A)-0.1% formic acid acetonitrile (B) as the mobile phase. The sample injection volume was 5 μl. The flow rate was set at 0.3 ml/min, with gradient elution procedures as: 0–13.00 min, 5 %∼100% B; 13.00–13.10 min, 100 %∼5% B; and 13.10–15.00 min, 5% B.

#### 3.3.3 Mass Spectral Condition

The ion source of HRMS was in ESI + positive ion mode by electrospray ionization. The ion source temperature was 550°C, the collision voltage was 500 V, the fragmentation voltage was 80 V, and the collision energies were 35 and 15 eV, respectively. Nitrogen was used as the atomizing gas, with auxiliary gases Gas1 and Gas2 at 55 PSI and curtain gas at 35 PSI. The first-stage mass spectrum scanning range was set at 80–1500 Da, and the IDA set the two-stage mass spectrum scanning to the eight highest peaks with the response value exceeding 100cps, and the sub-ion scanning range was set at 50–1,500 Da. Dynamic background subtraction (DBS) was set to reduce the interference. All data were analyzed with Peakview 2.0/Masterview 1.0 software.

#### 3.3.4 Data Processing

Data were acquired using Analyst TF 1.6 and were analyzed with Peakview 2.0/Masterview 1.0 software. The exact molecular formula within the mass deviation range of 5 × 10^–6^ was calculated. The specific compound types were determined by comparison with the chemical composition database of Qinbai (Radix Scutellariae) in the laboratory.

### 3.4 *In Vivo* Animal Experiments

#### 3.4.1 Culture of Mycoplasma Pneumoniae

MP (ATCC 15531) was purchased from American Type Culture Collection and was cultured in fetal bovine serum for 24 h before it was incubated in PPLO medium containing 20% fetal bovine serum and 10% yeast every 7 d for passage.

#### 3.4.2 Determination of Wogonin in Qinbai Extract by HPLC

Standard preparation: 1.05 mg of wogonin standard was dissolved in methanol to make the 0.105 mg/ml standard stock solution. One milliliter of this stand solution was placed in a 10-ml volumetric flask, diluted with methanol to a standard solution (10.5 μg/ml) before the solution was filtered through a 0.45 μm membrane.

Sample treatment: Three portions of 1 g Qinbai extraction were mixed with 10 ml of 70% methanol respectively, followed by ultrasonic extraction for 30 min, before which 70% methanol was added to make up for the mass, and the resulted mixture was centrifuged at 5,000 rpm for 30 min. The supernatant was taken and filtered.

#### 3.4.3 HPLC Chromatographic Conditions

Shimadzu LC-2030C3D was used for HPLC analysis. The chromatographic column was Diamond SIL C18 (2) (250 × 4.6 mm, 5 μm). The mobile phase was the mixture of acetonitrile (A) with 0.1% formic acid aqueous solution (B). The flow rate was set at 1.0 ml/min with column temperature maintained at 30°C. Sample injection volume was 20 μl, and UV wavelength was set at 280 nm. Elution procedures were: 0–5 min, 15 %∼33% A; 5–8 min, 33 %∼37% A; 8–25 min, 37 %∼50% A; and 25–30 min, 50 %∼55% A.

#### 3.4.4 Establishment of the Mycoplasma Pneumoniae Model in BALB/C Mice

Forty BALB/c mice, half male and half female, were randomly divided into a control group, a model group, an azithromycin group, a pentoxyverine group, and a wogonin group, with eight mice in each group. In addition to the control group, after ether anesthesia, mice in each group were given 20 μl of 10^6^ color change unit (CCU) MP by nasal drip once a day for a total of 3 d. The HPLC method was used to determine the content of wogonin in Qinbai extract and to determine the dosage of wogonin for mice. The wogonin group was treated with wogonin 74.27 μg/kg/d, the azithromycin group was given azithromycin 32 mg/kg/d, and the pentoxyverine group was given pentoxyverine 1.35 mg/kg/d. The drug was administered for 7 d consecutively. The control group and the model group were given the same dose of normal saline.

#### 3.4.5 Cough-Inducing Experiment

Mice in each group were placed one by one into an inverted 500 ml glass beaker with a cotton ball in it, and 0.5 ml ammonia water was taken with a 1 ml syringe and added dropwise onto the cotton ball, which was then quickly fastened to the beaker. The cough latency (the number of seconds from the time of the first cough after the addition of ammonia) was observed and recorded. The number of coughs within 2 min was observed and recorded.

#### 3.4.6 Immunohistochemical Experiment

The lung tissue of mice in each group was completely removed from the thoracic cavity, and the same part of the removed lung was embedded with an automatic paraffin embedding machine. The lung tissue was frozen in the refrigerator at −20°C for 4 h before it was sliced. After the tissues were cut into 5 μm thick sections, immunohistochemical reactions were performed, according to the manufacturer’s instructions. The distribution of TRPA1 expression in lung tissue of mice in each group was comparatively observed under the light microscope. Image J analysis software was used to analyze the images and calculate the average optical density (AOD) value of TRPA1 expression. SPSS 18.0 analysis software was used to analyze the data. AOD = integrated option density (IOD)/area.

#### 3.4.7 Western Blot Experiment

According to the instruction coming with the protein lysis kit and the protein detection kit, the tissue protein was extracted and the protein concentration was detected, and 5× loading buffer was mixed with the protein solution in a volume ratio of 1:4, with the loading amount of each histone at 50 μg. After the proteins were transferred to the PVDF membrane by 12% SDS-PAGE, the membrane was incubated overnight with rabbit anti-TRPA1 antibody at 4°C, and the goat antirabbit secondary antibody was blocked by ECL-developing solution for 4 min before being incubated at room temperature for 60 min. The gel imaging analyzer was used for imaging, and then the gray value of the target band was quantitatively analyzed by gel image processing software Image J. The target protein was standardized with β-actin protein to analyze the difference in protein changes.

#### 3.4.8 Detection of Transient Receptor Potential A1, Substance P, and CGRP mRNA Expressions by Real-Time PCR

The mice were sacrificed by dragging the cervical vertebra and the lung tissues were taken. The RNA of mice in each group was extracted according to instructions coming with the RNA extraction kit, and the RNA concentration was detected with a microplate reader. The expression of TRPA1 mRNA was amplified and detected according to the one-step reverse transcription PCR kit. The real-time PCR amplification procedure was as follows: the reaction was maintained at 37°C for 15 min, followed by the initial denaturation at 95°C for 10 min, and 40 cycles of denaturation at 95°C for 10 s, before the primer was annealed at 60°C for 30 s, and was maintained at 72°C for 30 s for the extension. The primer sequences are shown in Table 1.

**TABLE 1:** PCR primer sequence.

Primer	Sequence
TRPA1	Forward primer: 5′-GCG​GTT​GGG​GAC​ATT​GCT​GAG-3′
	Reverse primer: 5′-TGG​ATA​CAC​GAT​GGT​GGA​CCT​CTG-3′
SP	Forward primer: 5′-TTG​GTC​CGA​CTG​GTC​CGA​CAG-3′
	Reverse primer: 5′-GAACTGCTGADDCTTGGGTCTTC-3′
CGRP	Forward primer: 5′-AGT​GAA​GAA​GAA​GTT​CGC​CTG​CTG-3′
	Reverse primer: 5′-CCT​CCT​GCT​CTT​CCT​CCT​GCT​C-3′
β-actin	Forward primer: 5′-GAC​GGC​CAG​GTC​ATC​ACT​ATT​G-3′
	Reverse primer: 5′-AGG​AAG​GCT​GGA​AAA​GAG​CC-3′

### 3.5 Statistical Methods

The data of immunohistochemistry, real-time PCR, and Western blot were analyzed using SPSS 18.0. The data were expressed as ‾x ± s and the difference were statistically significant if *p* < 0.05.

## 4 Result

### 4.1 Transient Receptor Potential A1 Protein is Coupled With CM5 Chip

**FIGURE 1:**
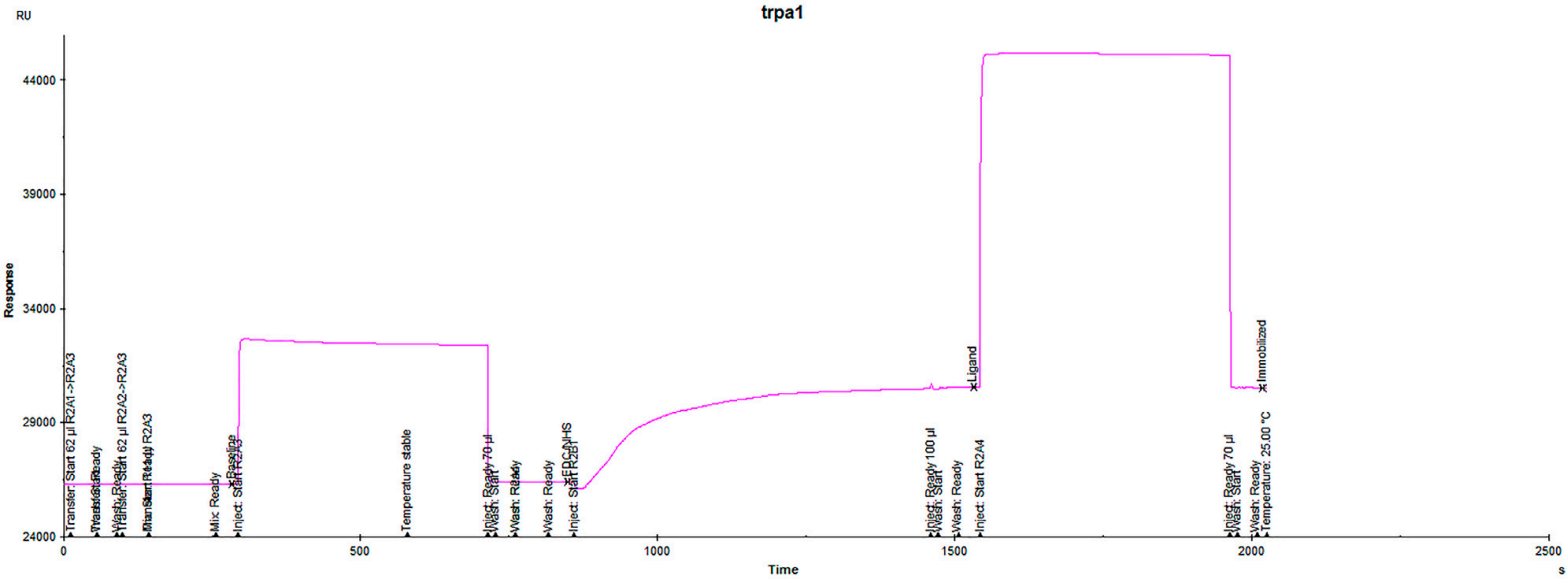
TRPA1 protein couple with CM5 chip in the Biacore™ experiment.

The enrichment effect was best when the pH of sodium acetate solution was 4.0, which was selected as the enrichment condition for this experiment. As shown in the Figure 1, the response signal value reached 4230.6 RU when the protein was coupled to the chip, showing that the protein had a good binding effect with the chip, which met the requirements for subsequent experiments. (Figure 1).

### 4.2 Qinbai Binds to Transient Receptor Potential A1 Protein

**FIGURE 2:**
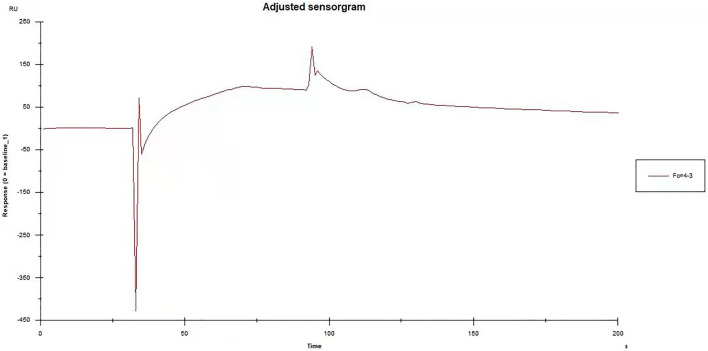
Binding of TRPA1 to Baiqingfei concentrated pellet by Biacore™ assay.

Figure 2 shows the binding response curve of Qinbai extract solution and TRPA1 protein. As shown in the figure, the binding value of the response curve reached 99.0 RU, indicating that Qinbai extract combined with TRPA1 protein well *in vitro*. (Figure 2).

### 4.3 Fishing Results of Qinbai and Transient Receptor Potential A1 Protein Ligand

**FIGURE 3:**
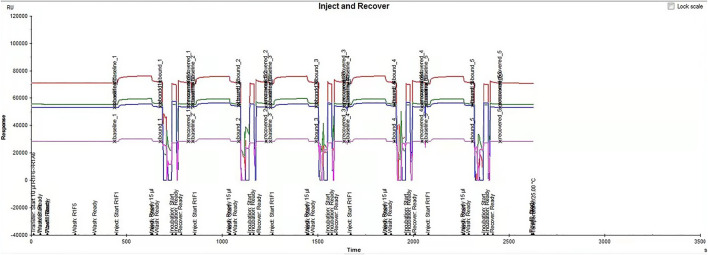
Qinbai obtained fishing results with TRPA1 protein ligand by the Biacore™ fishing technique.

Figure 3 showed the process of binding and dissociation of Qinbai and TRPA1 protein in ligand fishing and enriched the components from the Qinbai as much as possible that can be combined with TRPA1 protein. A total of 20 cycles were set, and each cycle was repeated five times. The results showed that the peak-binding value of Qinbai and TRPA1 protein was in the range of 60,000–75,000 RU. The curve of the binding process was significantly increased, the binding value of the dissociation process was significantly decreased and then returned to the baseline, and then the next cycle was conducted, indicating that the conjugation had been successfully dissociated and recovered. (Figure 3).

### 4.4 ESI-MS2 Mass Spectrometry Data of Wogonin in the Qinbai Recovery Sample

**FIGURE 4:**
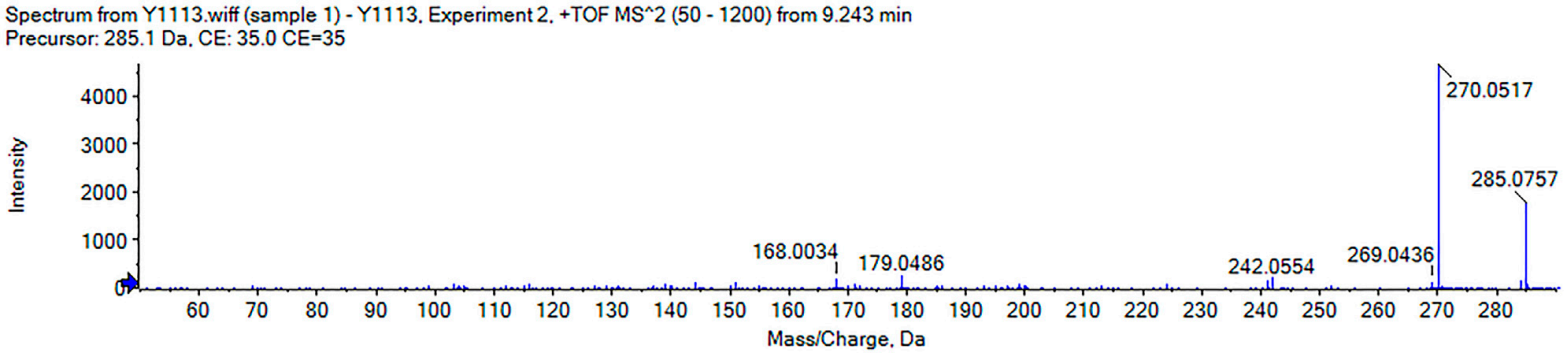
Sample was analyzed by mass spectrometry to obtain an ESI-MS2 mass spectrum of wogonin.

The mass spectral analysis revealed the formation of proton ions in positive ionization mode. Its quasi-ionic peak, m/z 285.075 45 [M + H]^+^, is [M + H]^+^ = 285, and M = 284. In a further mass spectrometric cleavage process, one molecule of the methyl group is removed by the positive ion peak to form a characteristic fragment ion m/z 270 [M + H-CH_3_]^+^. Based on the elemental composition analysis, the molecular formula of the compound is C_16_H_12_O_5_ with an error of −1 × 10^−6^. Through data comparison on the main cleavage characteristics of wogonin positive ions, the harvested samples were identified as wogonin. (Figure 4).

### 4.5 HPLC Assay Results

**FIGURE 5:**
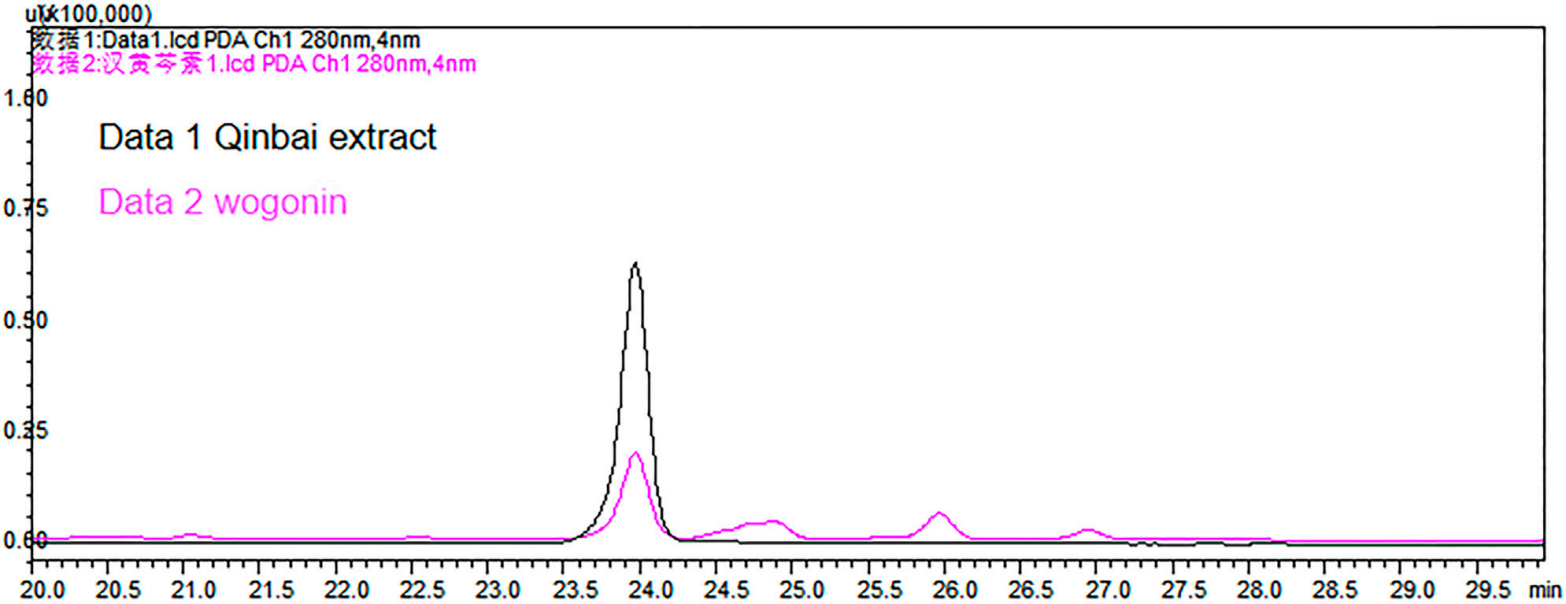
HPLC chromatograms of Qinbai extract and wogonin.

HPLC results showed that the wogonin content in Qinbai extract was 31.74 μg/g. According to the dosage equivalent to that in the mouse experiment, the dosage of wogonin was 74.27 μg/kg (Table 2; Figure 5).

**TABLE 2:** Experimental results of determination of wogonin in Qinbai extract by HPLC.

Sample name	Quality	Volume (ml)	Retention time	Peak area	Concentration (μg/ml)	Content (μg/g)
Wogonin	1.05	100	23.970	857683	10.5	
Qinbai1	1.0386	10	23.971	267595	3.28	31.54
Qinbai2	1.0327	10	23.967	265611	3.25	31.49
Qinbai3	1.0337	10	23.970	271910	3.33	32.20
Average value						31.74

### 4.6 Results of Cough-Inducing Experiment

The results of cough induction experiments showed that wogonin could effectively reduce the number of coughs in MP-infected cough mice. Compared with that of the control group, the cough latency was shortened in the model group (*p* < 0.01), and the number of cough episodes within 2 min increased in the model group (*p* < 0.01), indicating that the model was successfully established. Compared with the model control group, the cough latencies of the azithromycin group, pentoxyverine group, and wogonin group were longer (*p* < 0.01), and the number of coughs within 2 min declined (*p* < 0.01). Among them, the number of coughs within 2 min decreased significantly in the wogonin group, and the antitussive effect of the wogonin group was better than that of the azithromycin group. At present, azithromycin side effects such as resistance are the main problems during clinical treatment. Pentoxyverine has antitussive effect but does not have the antimycoplasma effect. Qinbai can make up for the shortcomings of the aforementioned drugs and has the potential as a new treatment (Table 3).

**TABLE 3:** Antitussive effect of wogonin on MP infection in mice (*n* = 8, ‾x ± s), ^*^ *p* < 0.01, compared with the model group. **p < 0.01, compared with the control group.

Group	Dose	Cough latency/s	Cough frequency in 2 min
Control	‑	6.94 ± 1.23	23.71 ± 7.09
Model	‑	2.40 ± 0.74**	43.13 ± 6.66**
Azithromycin	32 mg/kg/d	4.28 ± 0.65*	24.38 ± 9.89*
Pentoxyverine	1.35 mg/kg/d	4.58 ± 0.82*	19.13 ± 4.80*
Wogonin	74.27 μg/kg/d	4.30 ± 1.29*	21.63 ± 9.69*

### 4.7 Determination of Transient Receptor Potential A1 Protein Expression by Immunohistochemistry

**FIGURE 6:**
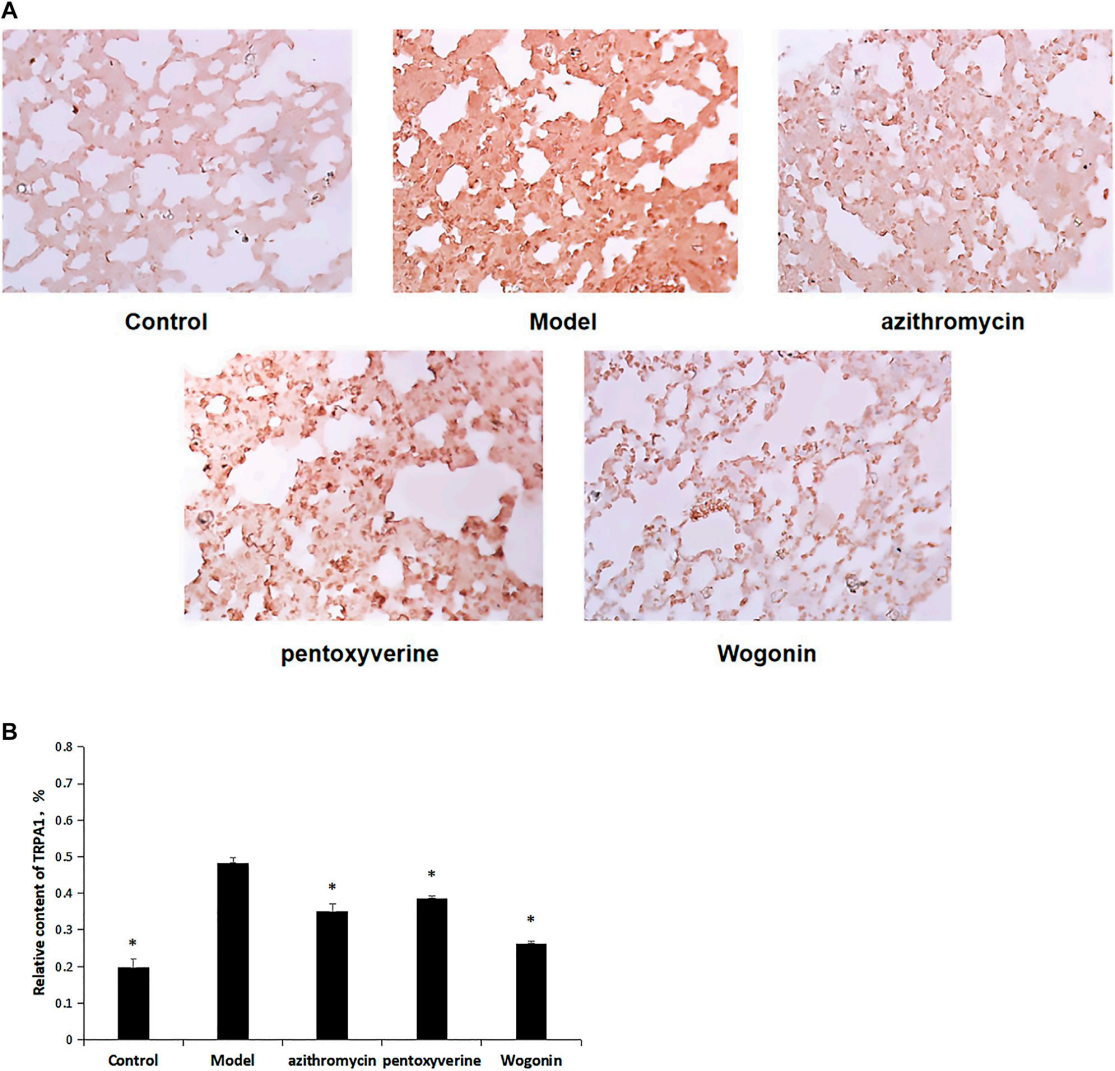
**(A)** Effect of wogonin on the expression of TRPA1 protein in lung tissue of mice infected with MP detected by immunohistochemistry. **(B)** Determination of AOD value of TRPA1 protein expression in lung tissue of mice infected with MP after administration of wogonin by immunohistochemistry, ^*^ *p* < 0.01.

As shown in Figures 6A,B, immunohistochemical results showed that compared with that for the model group, the TRPA1 protein in the control group was less and sparsely distributed, and the AOD value was lower. The difference between the two groups was statistically significant (*p* < 0.01), suggesting that the TRPA1 protein expression in cough mice after MP infection increased. Compared with that for the model group, the positive expression of TRPA1 in each administration group was significantly reduced, and the AOD value expression of TRPA1 in the wogonin group was the lowest (*p* < 0.01), suggesting that TRPA1 protein was downregulated *in vivo* after wogonin administration. (Figures 6A,B).

### 4.8 The Expression Levels of Transient Receptor Potential A1, Substance P, and CGRP Proteins Were Detected by Western Blot

**FIGURE 7:**
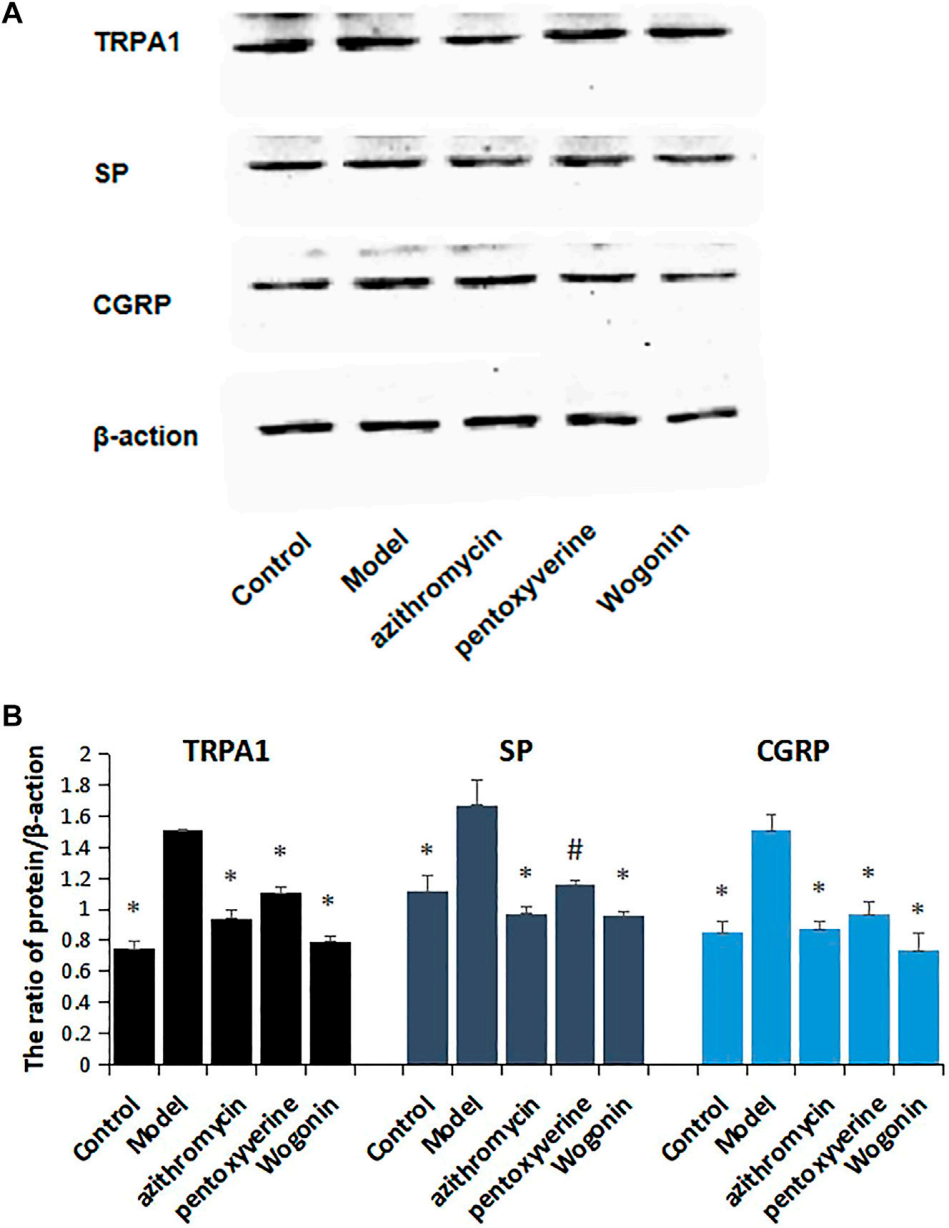
**(A)** Effects of wogonin on the expression of TRPA1, SP, and CGRP proteins in lung tissue of mice infected with MP. **(B)** Analysis of TRPA1, SP, and CGRP protein expression data in the lung tissue of MP-infected mice after administration of wogonin, ^*^ *p* < 0.01, ^#^ *p* < 0.05, compared with the model group.

As shown in Figures 7A,B, with the expression of β-actin set as one, the protein expression levels of TRPA1, SP, and CGRP in the control group were decreased, compared with those of the model group, and the differences were statistically significant (*p* < 0.01). Compared with those of the model control group, the expression levels of SP and CGRP proteins in each administration group were decreased, and the differences were statistically significant (*p* < 0.01 and *p* < 0.05). (Figures 7A,B).

### 4.9 Effects of Wogonin on Expressions of Transient Receptor Potential A1, Substance P, and CGRP mRNA in Lung Tissue

**FIGURE 8:**
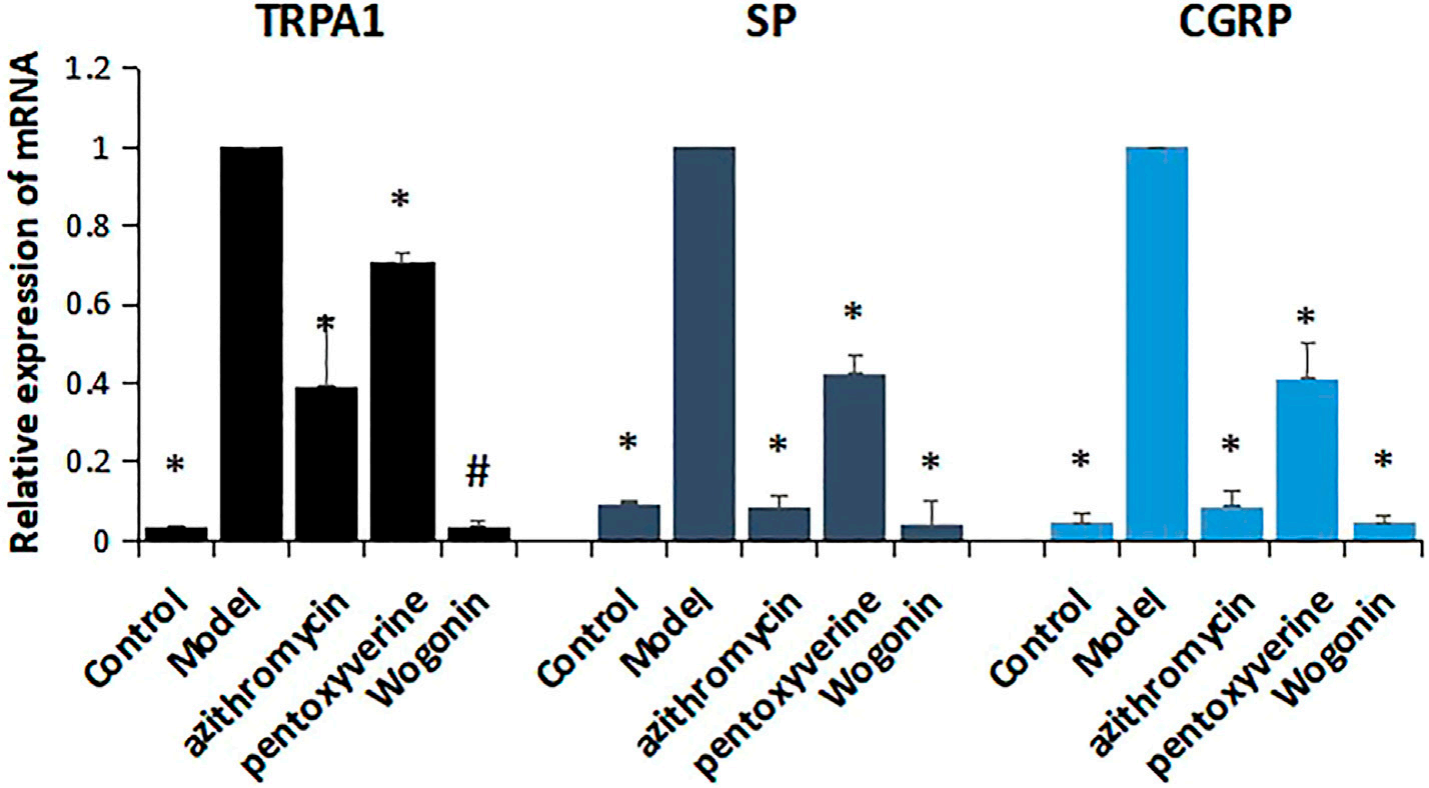
Effect of wogonin on the expression of TRPA1, SP, and CGRP mRNA in lung tissue of mice infected with MP, ^*^ *p* < 0.01, ^#^ *p* < 0.05, compared with the model group.

Compared with those of the model group, the expression levels of TRPA1, SP, and CGRP mRNA in control group were decreased, and the differences were statistically significant (*p* < 0.01). Compared with those of the model group, the expressions of TRPA1, SP, and CGRP mRNA in the wogonin group and the positive control group were decreased, and the differences were statistically significant (*p* < 0.01 and *p* < 0.05). The effect of wogonin was superior to that of the azithromycin group and pentoxyverine group. The results showed that wogonin could significantly reduce the expressions of TRPA1, SP, and CGRP in cough mice infected with MP. (Figure 8).

## 5 Discussion

MP is mainly transmitted through the respiratory tract. The literature has shown that among non-bacterial pneumonia, nearly half of respiratory tract infections are caused by MP (Jin et al., 2012). Cough is the main respiratory symptom caused by MP infection, and persistent dry cough or cough with small amount of sputum is one of its main characteristics. Among traditional Chinese medicines, Qinbai is the first new Chinese medicine for the treatment of mycoplasma pneumonia in children and has been proven to have a good curative effect in the treatment of mycoplasma pneumonia. Wogonin is the main active component of Qinbai. Studies have shown that wogonin has the pharmacological effects of antivirus, antitumor, and anti-inflammation, and wogonin can mediate mitochondrial injury-related cell apoptosis by inducing the production of ROS in fibroblast-like synovial cells and then activating the p38MAPK signaling pathway (Wang et al., 2017). Wogonin may interfere with the inflammatory response of macrophages by reducing the production of ROS in macrophages, reducing the nuclear localization of NF-κB, and further weakening the gene transcription of inflammation-related molecules regulated by NF-κB (Zhang et al., 2019). However, there are few studies on wogonin for the treatment of cough after infection. This study focused on the mechanism of wogonin in inhibiting cough potential protein and treating cough after MP infection.

In this study, the strong specific binding of TRPA1 protein to Qinbai *in vitro* was detected, with the binding value of 99.0 RU, demonstrating a stable binding of TRPA1 to Qinbai *in vitro*. The active components of Biacore™ angling were analyzed by mass spectrometry and identified as wogonin. The application of Biacore™ in the field of traditional Chinese medicine has gradually become a popular research topic. Xu et al. (2020)demonstrated the effective binding of wogonin and TGF-β1 protein *in vitro* through the affinity test results of Biacore™, and the effective binding component was wogonin. It was reported that earthworm and TGF-β1 protein have a good binding effect *in vitro*, and that earthworm can inhibit the progression of pulmonary fibrosis *in vivo* by inhibiting the expressions of TGF-β1 and α-SMA protein (Wang et al., 2019).

In this experiment, the MP pneumonia model was established by nasal drip of MP bacterial solution. The lung tissue of mice was taken to extract proteins and mRNA. The cough potential protein TRPA1 and the expression levels of SP and CGRP proteins were detected by immunohistochemistry, Western blot, and real-time PCR experiments. The results of this experiment showed that compared with those of the model control group, the protein and mRNA expressions of TRPA1, SP, and CGRP in the wogonin administration group were decreased. The afferent nerves of airway related to cough reflex mainly include Aδ fibers and C fibers. C fiber is distributed almost throughout the respiratory system, from the upper respiratory tract to the lower respiratory tract, and then to the alveolar. TRPA1 is widely distributed on C fibers and plays an important role in respiratory diseases. It has been found that the TRPA1 gene knockout mouse asthma model has reduced inflammatory factors and airway hyperresponsiveness (Tamura et al., 2008). Xu et al. (2018) found that the TRPA1 channel plays an important role in PM2.5-induced lung inflammation, and activation of the TRPA1 channel can aggravate asthma symptoms. TRPA1 can be activated by a variety of exogenous stimuli, such as cinnamic aldehyde, allicin, acrolein, mustard oil, and cannabinoid. TRPAI can also be activated by inflammatory mediators such as the lipid peroxidation product 4-HNE and prostaglandin J2 (Fajer and Ahmed, 2019). The activation of TRPA1 can cause Ca2^+^ influx, causing nerve endings to release tachykinin, CGRP, and SP, which act on relevant effector cells of the respiratory tract and cause local axonal reflex such as bronchoconstriction, protein exudation, and inflammatory reaction. CGRP and SP are neuropeptide mediators (Lee et al., 2003), and neuropeptides can directly stimulate cough receptors, reduce cough threshold, and increase the sensitivity of the airway to various stimuli, thereby directly causing acute and chronic cough. TRPA1 controls the release of CGRP and SP to induce inflammation, and the released CGRP and SP can promote the degranulation of mast cells to release a large number of inflammatory mediators, such as histamine, kinin, IL-6, and IL-8 (McKemy et al., 2002). SP can increase vascular permeability and play a pro-inflammatory role. CGRP can promote the release of primary afferent fiber terminals from SP, enhance the biological effects of SP, and promote the transmission of pain information and the occurrence of inflammatory reaction (Ewing T and Kuntz, 1997). This indicates that activation of TRPA1 can release inflammatory factors and enhance cough response, and inhibition of TRPA1 can be used as a potential therapeutic target for cough after MP infection. In summary, wogonin might inhibit the activation of TRPA1, thereby reducing the release of SP and CGRP, increasing the cough threshold, reducing the sensitivity of the airway to a variety of stimuli, and relieving the cough symptoms after MP infection.

Herbal medicine compound has many components, and there may be interactions between multiple components. The study of a single component can reveal the effective substance component of a compound drug and provide theoretical support for new drug research.

The aforementioned studies have demonstrated that wogonin has a good cough suppression effect *in vitro* and *in vivo*. The expression levels of cough related factors TRPA1, SP, and CGRP before and after administration were analyzed, which further revealed the mechanism of Qinbai in the treatment of MP pneumonia and cough caused by MP infection. In this study, the interaction between drugs and target proteins was explored by using Biacore™, which provided a reference for the future study of the pharmacological action mechanism of the active ingredients in herbal medicine.

## Data Availability

The raw data supporting the conclusion of this article will be made available by the authors, without undue reservation.
